# Role of Otolaryngologists in the Treatment of Patients With Riboflavin Transporter Deficiency: A Case Report

**DOI:** 10.7759/cureus.36312

**Published:** 2023-03-17

**Authors:** Mohammad I Alasqah, Bshair Aldriweesh, Waleed A Alshareef, Muataz H Alhashem, Ahmad Alammar

**Affiliations:** 1 College of Medicine, King Saud University, Riyadh, SAU; 2 Department of Otolaryngology, Head and Neck Surgery, King Saud University Medical City, Riyadh, SAU; 3 Department of Otorhinolaryngology, Head and Neck Surgery, King Fahad Specialist Hospital, Dammam, SAU

**Keywords:** hearing loss, stridor, riboflavin, vitamin deficiency, genetic mutation

## Abstract

Riboflavin transporter deficiency (RTD) is a rare genetic disorder that can have detrimental effects on the nervous system, causing progressive neurodegeneration. Here, we report the second case of RTD in Saudi Arabia. An 18-month-old boy presented to the otolaryngology clinic with six weeks history of progressive noisy breathing associated with drooling, choking, and difficulty in swallowing. Progressive regression of the child’s motor and communicative abilities was reported as well. Upon examination, the child had biphasic stridor, chest retractions, bilateral facial palsy, and hypotonia. The presence of an aerodigestive foreign body or congenital anomalies was excluded using bronchoscopy and esophagoscopy. Empirical high-dose riboflavin replacement therapy was initiated upon anticipation of diagnosis. Whole exome sequencing revealed a *SLC52A3* gene mutation, which confirmed the diagnosis of RTD. After a period of intensive care unit (ICU) admission with endotracheal intubation, the child’s general condition improved, and he was weaned off of respiratory support. Tracheostomy was avoided in this patient, as he responded to riboflavin replacement therapy. During the disease course, an audiological assessment revealed severe bilateral sensorineural hearing loss. He was discharged home on gastrostomy feeding owing to the risk of frequent aspiration, and he was regularly followed up by the swallowing team. The early initiation of high-dose riboflavin replacement appears to be of great value. The benefits of cochlear implants in RTD have been reported, but not fully established. This case report will increase awareness in the otolaryngology community about patients with this rare disease who might initially present to the clinic with an otolaryngology-related complaint.

## Introduction

Riboflavin, also known as vitamin B2, is a water-soluble vitamin that plays a significant role as a precursor for multiple enzymatic reactions in human cells [1]. It plays a role in the electron transport chain and metabolic processes of amino acid, glucose, and fatty acid [1]. An inadequate supply of riboflavin caused by *SLC52A3* mutation of the vitamin B2 protein transporter can have a detrimental effect on the nervous system, leading to progressive neurodegeneration, usually presenting as cranial nerve deficits, hearing loss, respiratory symptoms, muscle weakness, and feeding difficulties [2]. We report the second case of riboflavin transporter deficiency (RTD), a disorder also known as Brown-Vialetto-Van Laere (BVVL), in Saudi Arabia.

## Case presentation

An 18-month-old male toddler was brought to the otolaryngology clinic by his parent because of noisy breathing for six weeks. He was born full-term without any known medical conditions. The postnatal period was uneventful until two months before presentation, when he developed high-grade fever. Following the febrile episode, his parents observed a gradual evolution of choking and difficulty swallowing, which was associated with saliva drooling, occasional aspiration of liquids, and weight loss. His symptoms progressed as he developed poor sucking and oral control, his smile was abnormal, and he became less interactive. Finally, he developed noisy breathing aggravated by crying and increased during feeding. There was no history of cyanosis, witnessed foreign body ingestion, or intubation. Developmental milestones were initially normal and then regressed, which was most pervasive in his language skills. He was sitting at seven months, crawling around nine months, and standing with support at one year. At the time of presentation, the patient could only stand with support and say only two words. His parents were consanguineous third-degree relatives, and his older siblings were healthy. There were no known genetic disorders in the family.

Upon admission to the pediatric and airway surgery otolaryngology clinic, he presented with mild respiratory distress with biphasic stridor, tracheal tugs, and subcostal retractions. He had an apathetic face without dysmorphic features, a left eye squint, and bilateral facial nerve palsies with lagophthalmos. Air entry was equal bilaterally with transmitted sounds, and heart auscultation was normal without murmurs. He had hypotonia, and his abdomen was soft and lax.

Direct laryngoscopy and bronchoscopy (DLB) and esophagoscopy showed mildly edematous arytenoid mucosa, normal passive mobility of the vocal cords, normal subglottic and distal airway, no clefts or deep interarytenoid groove, normal esophageal mucosa, and no foreign body. Swallowing assessment revealed a significant reduction in laryngeal elevation (almost absent swallowing reflex), oral pharyngeal dysphagia, and incomplete airway protection. The patient was placed on a high-flow nasal cannula (HFNC) and nasogastric tube feeding.

Brain and spine MRI displayed smooth enhancement of the cauda equina nerve roots, associated with enhancement of multiple cranial nerves, probably post-viral or autoimmune polyneuropathy. Electroencephalogram and cerebrospinal fluid analyses were normal. A nerve conduction study and electromyography of the right upper and lower extremities showed electrodiagnostic evidence of sensorimotor, predominantly axonal, polyneuropathy with features of ongoing denervation in the distal right upper extremity muscles. Electrocardiograms and echocardiograms were normal. Bone marrow aspiration revealed no pathological abnormalities. Urine organic acids test showed a high peak of ethylmalonic acid, which was repeated and confirmed.

Empiric intravenous immunoglobulin and methylprednisolone therapies were administered with no clinical improvement. Empiric riboflavin replacement (20 mg/kg) was initiated because of a high index of RTD suspicion. Four days later, whole exome sequencing confirmed the diagnosis of BVVL syndrome 1, as it revealed a *SLC52A3* gene mutation. The riboflavin dose was increased to 80 mg/kg/day, divided twice daily, along with coenzyme Q10 supplementation.

Notably, his stay was complicated by two episodes of apnea and cyanosis during saliva suction, requiring cardiopulmonary resuscitation. Following resuscitation, the patient was intubated for five days in the pediatric intensive care unit (PICU) and then changed to HFNC. A second life-threatening event occurred four days later, and the patient was re-intubated. A multidisciplinary team meeting was held involving pediatric neurology, otolaryngology, genetics, clinical pharmacy, and PICU teams to discuss the management course and whether tracheostomy was required at this stage. The decision was to allow medical management to control secretions before considering tracheostomy, which was a standby alternative. Initially, he received Botox injection in the salivary glands to control drooling. However, no significant improvements were observed. Subsequently, ipratropium nebulization was administered for 48 h, but it had no significant effect. Finally, the patient was initiated on sublingual atropine drops, which successfully reduced his drooling. Following gradual improvement in riboflavin replacement therapy, the child was successfully extubated after 15 days to HFNC and then to room air after 29 days.

On admission, his nutritional intake was through a nasogastric feeding tube. After four months, he had a gastrostomy tube. The patient adhered to long-term tube feeding and saliva swallowing training using traces of thick liquids and puree while maintaining good oral hygiene.

**Figure 1:**
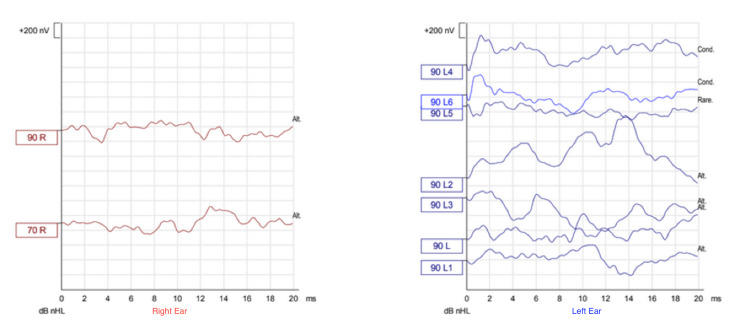
Auditory brainstem response Air-conduction chirp auditory brainstem response (broadband stimulus, not frequency specific). No response was recorded up to a maximum stimulation level of 90 dB nHL, bilaterally. dB nHL = decibel above normal hearing level. R = Right. L = Left. nV = Nanovolt. ms = Milliseconds.

**Figure 2:**
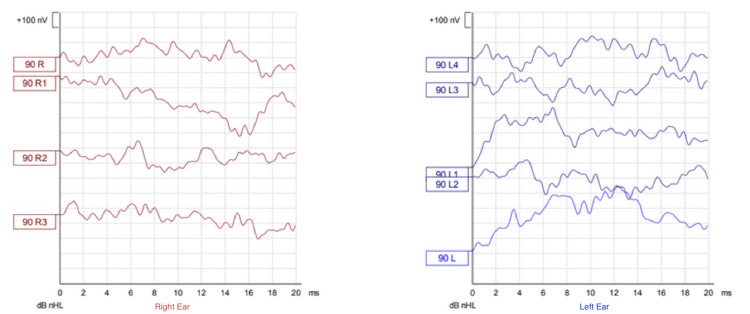
Auditory brainstem response Air conduction tone burst applied at 500 Hz. No response was recorded up to a maximum stimulation level of 90 dB nHL, bilaterally. dB nHL = decibel above normal hearing level. R = Right. L = Left. nV = Nanovolt. ms = Milliseconds.

**Figure 3:**
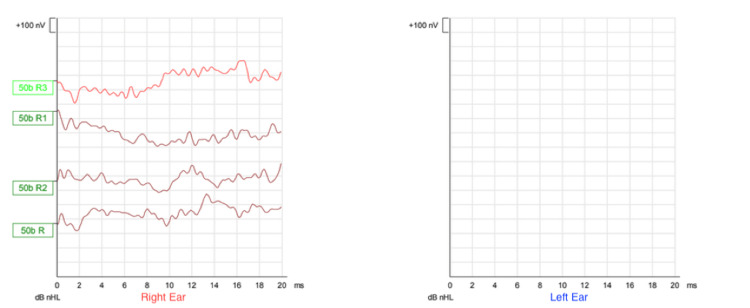
Auditory brainstem response Bone conduction chirp auditory brainstem response (broadband stimulus, not frequency specific). No response was recorded up to a maximum stimulation level of 50 dB nHL, bilaterally. dB nHL = decibel above normal hearing level. R = Right.  nV = Nanovolt. ms = Milliseconds.

**Figure 4:**
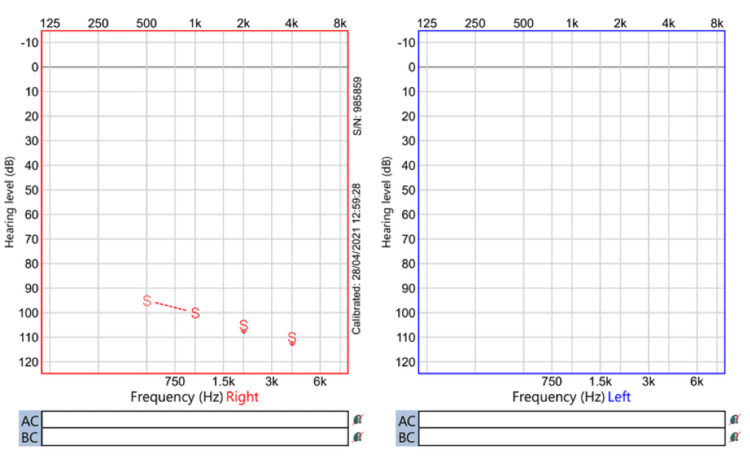
Visual reinforcement audiometry Unaided visual reinforcement audiometry using speakers. It shows severe to profound hearing loss in at least one ear. dB = decibel. k = kilos. Hz = Hertz.

**Figure 5:**
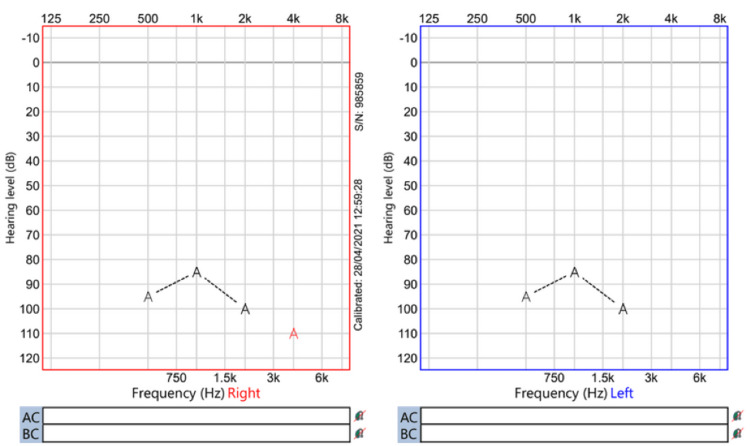
Visual reinforcement audiometry Aided visual reinforcement audiometry using speakers. It shows severe to profound hearing loss bilaterally. dB = decibel. k = kilos. Hz = Hertz.

Regarding audiological assessment, auditory brainstem response (Figures 1-3), distortion product otoacoustic emissions, and cochlear microphonic results showed severe bilateral auditory neuropathy; therefore, he was provided with two hearing aids (Phonak Naida v50 UP). Two months later, there was no improvement in his hearing (Figures 4-5), and the cochlear implant committee accepted him for sequential bilateral cochlear implants.

Currently, the child is two years old. At the last clinic visit, he showed substantial improvement in his facial expressions and social interactions, improved management of secretions, and significantly reduced drooling. His breathing pattern also improved, as he no longer had stridor, with improved muscle tone and weight gain, although oral feeding was still unsafe due to persistent oral dysphagia. The patient was scheduled to undergo a modified barium swallow.

## Discussion

In the past few years, progress has been made in the literature on RTD, a rare and challenging disease to identify without genetic testing. BVVL and Fazio-Londe syndromes are now recognized as RTD after discovering *SLC52A2* and *SLC52A3* gene mutations, which are the underlying pathologies in RTD type 2 (RTD2) and RTD type 3 (RTD3), respectively [2]. Hearing loss and muscle weakness are common presentations in both types [3]. RTD3, however, is characterized by more prevalent bulbar symptoms at the onset of the disease, and affected patients experience facial palsy and feeding difficulty. Our patient experienced drooling and swallowing difficulty, which ultimately led to gastrostomy tube placement, similar to reports of previous studies [2,3]. Both RTD2 and RTD3 typically present early in life, and the reported mean age of presentation was 3.0 and 5.6 years, respectively [2].

A unique aspect of our patient was that respiratory complaints in the form of breathing difficulty and stridor were reasons for seeking medical help. Although common, involvement of the respiratory system in patients with RTD is rarely an initial feature of this disease [2]. Respiratory complications are major contributing factors to morbidity and mortality in these patients [2]. Respiratory muscle weakness may necessitate temporary ventilatory support, and tracheostomy may be required during the clinical course [2]. However, despite improvement in respiratory function following therapy, some patients remain tracheostomy-dependent [4]. In our patient, however, tracheostomy placement was avoided. Medical management was sufficient to improve his respiratory muscle strength, and he was successfully weaned off a mechanical ventilator.

A similar case with confirmed *SLC52A3* gene mutation was reported in Saudi Arabia, but with more severe symptoms and older age at diagnosis. The patient additionally had vision loss, generalized tonic-clonic seizures, peripheral spasticity, and vertical nystagmus. He was bedridden and admitted to the hospital for a longer duration. Unfortunately, he died after the delayed diagnosis and his parents’ refusal of treatment [5].

Clinical suspicion of RTD warrants early empirical initiation of oral riboflavin replacement prior to genetic confirmation to ensure maximum benefit [2]. There is no consensus on the recommended dose of riboflavin replacement therapy, which varies among different reports between 7 and 60 mg per kg per day [2]. Riboflavin replacement therapy for children with RTD3 led to clinical improvement and stability in 80% and 20% of the cases, respectively [2]. Interestingly, in one case, the hearing threshold was at its best when measured immediately after riboflavin administration [6].

Cranial nerve palsies are common in this patient population, with involvement of cranial nerves VII-XII being the majority [2]. As part of the disease spectrum, sensory neural hearing loss is anticipated (present in 73% of patients) and requires vigilant care [2]. The characteristics of sensory neural hearing loss in these patients fall within the auditory neuropathy spectrum [7]. Our patient had a documented deterioration of his hearing level, which coincided with the onset of RTD.

Moreover, Anderson et al. (2018) reported four cases, one of which had a *SLC52A3* gene mutation with neurological and subjective hearing improvement after early riboflavin treatment; however despite using a hearing aid, her poor functional hearing persisted. Years later, she received a cochlear implant and finally improved despite the inability to assess speech because of her tracheostomy [8]. Hearing aids generally have limited benefits in these patients [7]. Fortunately, early riboflavin replacement has been demonstrated to improve the hearing threshold, especially in patients treated within 12-months following the onset of hearing loss [7]. Cochlear implant might be reserved for patients who fail to demonstrate improvement after 12 months of riboflavin treatment, especially if hearing aids are of limited value [7]. The cochlear implant committee accepted our patient to receive sequential bilateral cochlear implants.

## Conclusions

RTD is a rare and challenging condition that often presents with otolaryngology-related pathologies. Commonly encountered findings in this disorder related to otolaryngology are stridor and sensorineural hearing loss. The early initiation of high-dose riboflavin replacement seems to add great value to the treatment outcome. This case report intends to increase awareness in the otolaryngology community about patients who might initially present to the clinic with otolaryngology-related complaints.
